# Cultural Self-Construal and Sustainable Mental Health in Japan: The Role of Subjective, Objective, and Autonomous Selves

**DOI:** 10.3390/ijerph23020197

**Published:** 2026-02-03

**Authors:** Youngsun Yuk, Eiko Matsuda

**Affiliations:** 1 Graduate School of Human and Socio-Environmental Studies, Kanazawa University, Kanazawa 920-1192, Japan; 2 Department of Sociology, Toyo University, Tokyo 112-8606, Japan; eikom@toyo.jp

**Keywords:** cultural self-construal, subjective self, objective self, autonomous self, self-esteem, subjective well-being, psychological distress, insomnia symptoms

## Abstract

**Highlights:**

**Public health relevance—How does this work relate to a public health issue?**

- Psychological distress and sleep problems are common public health concerns in Japan and affect large numbers of people’s daily life, work, and education, with implications for everyday functioning and service needs. This study shows that these problems are linked to everyday, culturally shared ways of thinking about the self (self-construal), rather than being only individual or clinical issues.
- In particular, a socially reinforced tendency to view oneself through others’ evaluations (Objective Self; focus on others’ evaluation) is associated with higher levels of distress and sleep difficulties, while more internally guided ways of regulating the self (Subjective Self; acting from personal goals, and Autonomous Self; value-based self-regulation) are linked to better well-being.

**Public health significance—Why is this work of significance to public health?**

- This study is significant for public health because it identifies a culturally common and socially reinforced pattern (Objective Self) that is linked to higher levels of psychological distress and sleep problems. Because this pattern is widespread rather than limited to a small clinical group, its negative effects can accumulate at the population level.
- At the same time, the findings highlight culturally relevant protective patterns (Subjective Self and Autonomous Self) that are associated with better well-being. These patterns represent potential targets for mental health promotion and prevention strategies aimed at strengthening resilience across the population.

**Public health implications—What are the key implications or messages for practitioners, policy makers and/or researchers in the field of public health?**

- From a public health perspective, the findings suggest that improving mental health in Japan requires preventive approaches that address socially reinforced patterns of stress and emotional regulation, rather than relying only on individual clinical treatment.
- Population-based strategies—such as school, workplace, and community programs—can focus on reducing excessive social-evaluative pressure and supporting more internally guided coping and self-regulation. Such culturally sensitive approaches may help promote sustainable mental health across diverse social groups in contemporary Japan and support population-level mental health promotion policies.

**Abstract:**

Maintaining sustainable mental health is an increasing societal challenge in Japan, where psychological distress and sleep problems have become major public health concerns. This study examined how three culturally grounded dimensions of self-construal—Subjective Self (SS), Objective Self (OS), and Autonomous Self (AS)—relate to both positive and negative indicators of psychological adjustment among Japanese adults. This study aimed to examine whether internally guided forms of self-regulation (SS and AS) function as psychological resources, whereas externally guided self-regulation (OS) operates as a potential vulnerability factor in a culturally tight social context. By simultaneously examining multiple indicators of adjustment, this research clarifies how culturally shared self-regulatory patterns are linked to distress and sleep difficulties that affect large segments of the population. From a public health perspective, the findings highlight socially reinforced risk and protective patterns that can inform population-level prevention and mental health promotion in settings such as schools, workplaces, and communities, rather than relying solely on individual clinical intervention. These results underscore the importance of integrating cultural psychology into public health frameworks aimed at promoting sustainable mental health in contemporary and increasingly diverse social environments.

## 1. Introduction

Cultural self-construal refers to culturally shaped models of the self that guide how individuals interpret their relationships with others and position themselves within the social world. Markus and Kitayama [1] originally proposed the influential distinction between independent and interdependent self-construals, arguing that Western cultural ecologies promote an autonomous model of the self, whereas East Asian cultural ecologies foster a relational and socially embedded self. Although this framework has shaped decades of research in cognition, emotion, and motivation, subsequent scholarship has pointed out that it does not fully capture the diversity of culturally grounded selves observed across different societies.

Recent theoretical developments highlight that interdependence is not a homogeneous construct but manifests in multiple culturally specific forms [2]. For example, East Asian contexts often promote self-effacing interdependence grounded in emotional restraint; Arab ecologies foster honor-based interdependence; Latin American cultures encourage expressive interpersonal styles; and South Asian contexts rely on argumentative relational strategies. These perspectives align with the eco-cultural model, which conceptualizes cultural selfways as dynamic adaptive systems shaped by reciprocal interactions among ecological conditions, social institutions, and psychological processes [2]. From this viewpoint, cultural models of self are not static value orientations but evolving psychological patterns shaped through ongoing person–culture transactions.

Importantly, this framework suggests that cultural models of the self not only shape interpersonal tendencies but also regulate core psychological mechanisms, including emotion regulation, stress reactivity, and physiological arousal. Building on this perspective, the cultural self-construal perspective has been shown to systematically influence psychological well-being and mental health by shaping culturally normative patterns of emotion regulation and stress-related psychological processes [3,4]. These dynamics are especially relevant in contemporary Japan, where interpersonal sensitivity and emotional restraint are strongly emphasized [5].

Japan is currently facing significant public health concerns, including rising psychological distress, depression, anxiety, and chronic stress [6,7,8]. These problems reduce individual functioning and impose substantial social and economic costs, such as decreased productivity, increased occupational burden, and reduced social participation [9,10]. Sleep problems—including insufficient sleep and insomnia—are particularly severe, with Japan reporting among the highest global prevalence rates [10,11]. Insufficient sleep heightens worry, rumination, and emotional reactivity, while stress further disrupts sleep, creating mutually reinforcing cycles that undermine psychological stability [10,11,12]. From a sustainability perspective, identifying cultural psychological mechanisms that operate as resources or risk factors is essential for promoting sustainable mental health and social well-being [2]. In the present study, we use the term cultural psychological mechanisms to refer to culturally normative self-regulatory processes that shape how individuals experience, regulate, and respond to emotions and social stress in everyday life [1,13]. In the Japanese context, these mechanisms are characterized by heightened sensitivity to social evaluation, strong norms of emotional restraint, and the internalization of external expectations, which jointly influence patterns of emotion regulation and stress reactivity [14,15,16]. When internalized through socialization, these culturally normative behavioral patterns function as cultural psychological mechanisms by shaping habitual emotion regulation strategies, self-evaluative processes, and stress reactivity. Over time, such mechanisms influence individuals’ capacity to maintain psychological well-being while managing cumulative stress. In addition, sustainable mental health is conceptualized not as the absence of psychological symptoms alone, but as a dynamic balance between positive psychological resources and vulnerability to stress-related difficulties over time [17,18,19]. In public health research, sustainable mental health refers to the capacity of societies and public health systems to prevent the accumulation of distress and sleep-related problems through preventive, population-based strategies, rather than relying solely on individual treatment after problems emerge. Specifically, sustainable mental health refers to the capacity to maintain self-esteem and subjective well-being while minimizing the accumulation of psychological distress and sleep-related problems under recurrent social and emotional demands. From this perspective, indicators such as self-esteem and subjective well-being represent adaptive psychological resources that support resilience and emotional stability, whereas psychological distress (GHQ-12) and insomnia symptoms (AIS) reflect cumulative strain and dysregulation associated with prolonged stress exposure. Cultural psychological mechanisms are therefore theoretically and empirically central to sustainable mental health, as they structure habitual patterns of emotion regulation and self-regulation that shape how positive and negative psychological states co-occur and unfold across time within a given cultural context. From a public health perspective, these problems highlight the importance of identifying culturally shared psychological patterns that function as population-level risk or protective factors, rather than focusing only on individual clinical symptoms.

### 1.1. Existing Research on Cultural Self-Construal and the SOA Model

Building on the broader cultural and public health context outlined above, recent research has increasingly emphasized within-culture variability in self-construal contexts. Han, Kim, and Inumiya [20] introduced the Subjective–Objective–Autonomous (SOA) model as an alternative to the traditional independent–interdependent distinction, arguing that East Asian cultural contexts contain multiple coexisting selfways that shape individuals’ thoughts, emotions, and behavior. The SOA model conceptualizes cultural self-construal along three dimensions—the subjective self (SS), the objective self (OS), and the autonomous self (AS)—each representing a distinct mode of self-regulation embedded within everyday social interaction. It is important to note that although the subjective self (SS) and objective self (OS) may appear conceptually similar to subjective self-awareness (SSA) and objective self-awareness (OSA), as proposed by Duval and Wicklund [21], these constructs differ fundamentally in their theoretical level and function. SSA and OSA refer to momentary states of self-focused attention, describing whether individuals attend to their internal experiences or adopt an external, evaluative perspective. In contrast, SS and OS are conceptualized as culturally socialized and relatively stable forms of self-construal that reflect how individuals habitually position themselves within social relationships over time [20,22]. Thus, whereas SSA/OSA capture attentional focus at the state level, SS and OS represent culturally grounded self-regulatory orientations, or selfways, shaped through long-term socialization.

The subjective self (SS) reflects an agentic orientation grounded in internal standards such as personal desires, values, and goals. Individuals high in SS tend to express themselves actively, take initiative in interpersonal situations, and rely heavily on self-generated intentions, tendencies that support self-efficacy, stable self-esteem, and positive affect. The objective self (OS), in contrast, reflects viewing the self from the perspective of others. Individuals high in OS regulate their behavior according to social norms, expectations, and relational obligations, and tend to be highly sensitive to external evaluation and interpersonal comparison. OS is therefore closely linked to emotional suppression and heightened vigilance toward others.

The autonomous self (AS) reflects action guided by internalized values and beliefs that remain relatively independent of immediate social pressure. Although autonomy has often been viewed as a prototypically Western trait, empirical research based on the SOA framework demonstrates that the autonomous self is meaningfully represented in East Asian contexts and is systematically associated with indicators of psychological adjustment, including self-esteem and self-regulatory functioning [20,22,23]. Although both the SS and the AS are grounded in internal standards, they differ significantly in the source and contextual embedding of self-regulation. SS reflects an agentic self that operates within social relationships and emphasizes initiative, influence, and leadership in interpersonal contexts. Its internal standards are activated and expressed in relation to others. In contrast, AS reflects a form of selfhood guided by internalized values and beliefs that function relatively independently of immediate social demands. Thus, while SS represents agency within social context, AS represents agency from social context, a distinction that is central to the SOA model [20,23].

It is important to clarify that, in the present study, AS refers to a psychological self-construal concerning human self-regulation, value internalization, and agency in social contexts. This conceptualization is distinct from the use of “autonomous self-awareness” in artificial intelligence and robotics research [24,25] where autonomy typically denotes system-level independence or control rather than human psychological experience, motivation, or emotion.

Empirical studies have shown that Japanese adults typically score higher on OS than on SS or AS [20,22,23,26,27]. This pattern reflects long-standing cultural norms that emphasize modesty, emotional restraint, and sensitivity to others’ evaluations [3,28].

In the Japanese context, cultural desirability is closely linked to self-regulatory tendencies that promote interpersonal harmony and social appropriateness. According to cultural psychological research, behaviors such as emotional restraint, modest self-presentation, and heightened sensitivity to others’ expectations are commonly regarded as desirable and socially appropriate in Japan [2,14,29]. These traits are reinforced through everyday socialization processes, including family practices, educational settings, and workplace norms, where individuals who align with collective expectations are more likely to receive social approval and relational inclusion [1,13,28].

Taken together, such norms encourage individuals to internalize external expectations and to regulate emotional expression—especially in public settings—thereby reinforcing a predominantly OS-oriented mode of self-regulation. However, while OS may facilitate social harmony, prior work suggests that excessive reliance on OS can produce psychological costs, including increased physiological arousal, elevated stress reactivity, and impaired emotional well-being [3,28,30]. These findings raise important questions about how OS functions in contemporary Japan and whether its cultural desirability aligns with its psychological consequences.

In contrast to Japan, Korean studies have examined the SOA model more extensively, consistently showing that SS and AS predict higher well-being, whereas OS predicts lower well-being and greater emotional suppression. Yet systematic research in Japan remains limited, particularly studies that simultaneously assess all three SOA dimensions in relation to multiple indicators of psychological adjustment (self-esteem, subjective well-being, psychological distress, and sleep problems). Addressing this empirical and theoretical gap is a central aim of the present study.

### 1.2. The SOA Model, Psychological Adjustment, and Sustainable Mental Health

Psychological adjustment comprises both positive and negative components. Positive indicators—such as self-esteem and subjective well-being—represent internal psychological resources that support self-evaluation stability, emotional balance, and overall life satisfaction [31,32,33]. These indicators also contribute to broader social sustainability by enabling individuals to participate effectively in social and occupational contexts [34]. In contrast, negative indicators—such as psychological distress and sleep problems—reflect psychological vulnerabilities that undermine long-term well-being. Psychological distress captures emotional instability linked to stress, anxiety, depressed mood, and sensitivity to negative evaluation [35,36,37], whereas sleep problems, including insomnia symptoms, represent physiological manifestations of accumulated psychological strain and are considered a major public health concern in Japan [10,11,12]. Chronic emotional suppression, rumination, and heightened physiological arousal may further contribute to self-reinforcing cycles that impair both mental and physical functioning [3,30,37].

Taken together, these four indicators provide a comprehensive framework for examining the dual functions of cultural self-construal. Within the SOA framework, prior research suggests that these indicators map onto theoretically meaningful pathways of psychological adjustment. As Cross et al. [32] argue, agency-based self-views promote well-being even in collectivistic cultural contexts, a pattern reflected in the role of SS and AS. Individuals high in SS are more likely to express themselves openly, rely on internal standards in social contexts, and adopt problem-focused coping strategies—patterns associated with greater self-efficacy and enhanced well-being [23,38,39,40]. Likewise, empirical findings show that individuals high in AS maintain stronger self-consistency and engage in value-based, flexible emotion regulation, processes linked to stable subjective well-being and reduced stress reactivity [4,40,41].

By contrast, a growing body of research conceptualizes OS as a culturally embedded risk factor. Individuals high in OS tend to depend on external evaluations, suppress emotional expression, and engage in self-critical or ruminative thought patterns—tendencies associated with heightened stress reactivity and greater vulnerability to psychological distress [39,40]. Furthermore, studies on Japanese interpersonal norms indicate that emotional restraint and the maintenance of relational harmony are strongly emphasized culturally [5,26,27,42]. Such norms may reinforce OS-related regulatory tendencies, which have been linked to sleep disruption and increased insomnia risk among Japanese adults [9,10,11]. Overall, these patterns suggest that SS and AS function as cultural psychological resources, whereas OS may operate as a culturally amplified vulnerability factor. This theoretical framework provides the foundation for the hypotheses tested in the present study.

### 1.3. Present Study and Hypotheses

Previous experimental studies on objective self-awareness (OSA), conducted primarily in Western contexts, have reported mixed findings regarding self-esteem. Depending on situational conditions and the congruence between perceived performance and salient standards, heightened self-focus has been shown to increase self-criticism and lower self-esteem, facilitate self-regulation and enhance self-evaluation, or exert no significant effect. These findings suggest that OSA functions as a situational and momentary evaluative state whose psychological consequences are contingent on contextual cues. Importantly, however, OSA has typically been conceptualized as a transient attentional focus rather than as a culturally socialized mode of self-regulation.

Accordingly, it remains unclear whether findings from Western OSA research—largely based on transient manipulations of self-focus—can be directly generalized to cultural contexts such as Japan, where sensitivity to others’ evaluations and self-regulation based on external standards are chronically reinforced through socialization.

In contrast, prior studies conducted in Korea using the SOA framework provide a more detailed empirical basis for the proposed hypotheses by elucidating the psychological mechanisms associated with each self-construal type. Research has consistently shown that the Objective Self (OS), characterized by chronic sensitivity to others’ evaluations and the internalization of external standards, is associated with lower self-esteem [20,23], greater emotional suppression [26,27], and heightened psychological distress [22,23]. This pattern appears to reflect a self-regulatory tendency toward self-criticism and externally guided self-evaluation, which promotes vigilance to social norms but also increases vulnerability to psychological strain. Empirical findings further indicate that individuals high in OS are more likely to rely on suppressive or avoidant coping strategies, such as denial, and report lower happiness and interpersonal relationship satisfaction [38].

By contrast, the SS and AS have been consistently linked to more adaptive patterns of psychological adjustment. Individuals high in SS tend to rely on internal standards, exhibit greater self-efficacy, and adopt problem-solving and help-seeking coping strategies, which are associated with higher subjective well-being and interpersonal satisfaction [22,38]. Similarly, AS is characterized by value-based self-regulation, self-consistency, and relative independence from immediate social pressure, and has been shown to support stable self-esteem and flexible emotion regulation in East Asian contexts [20,22,23]. Taken together, these findings suggest that SS and AS function as psychological resources, whereas OS operates as a vulnerability factor, providing cumulative empirical justification for the hypotheses proposed in the present study.

Turning to the Japanese context, research in Japan has consistently demonstrated that Japanese adults score higher on OS than on SS or AS [20,22,23,26,27]. This creates an important theoretical question: if OS is culturally normative and socially reinforced in Japan, does it function as a cultural resource that supports adjustment or as a psychological risk factor? Although cultural-fit perspectives propose that alignment with dominant social norms may foster well-being, other evidence suggests that the interpersonal vigilance and emotional restraint associated with OS may increase psychological strain. Thus, whether OS is adaptive or costly within the Japanese context remains unclear.

The present study addresses this issue by examining how the three SOA dimensions—SS, OS, and AS—relate to four key indicators of psychological adjustment among Japanese adults: self-esteem, subjective well-being, psychological distress, and insomnia symptoms. By simultaneously assessing both positive and negative indicators within a single cultural context, this study provides a more comprehensive test of the dual pathways through which cultural selfways may support or undermine mental health in a culturally tight society that emphasizes harmony and emotional restraint.

Based on theoretical accounts of self-construal and prior findings in East Asian contexts, we formulated the following hypotheses:

**H1.**  *SS and AS will be positively associated with self-esteem and subjective well-being*.

**H2.**  *OS will be negatively associated with self-esteem and subjective well-being*.

**H3.**  *OS will be positively associated with psychological distress and sleep problems, whereas AS will be negatively associated with these indicators*.

## 2. Materials and Methods

### 2.1. Participants and Procedure

Data were collected through a web-based survey administered between June and August 2024. Participants consisted of two groups: university students and community-dwelling adults (hereafter “working adults”).

University students were recruited from psychology-related courses at a private Japanese university. After each class session, instructors provided a URL to the online questionnaire, and students accessed the survey using their personal devices. Working adults were recruited through multiple outreach channels, including LINE contacts, posts on social networking platforms (e.g., Facebook), and email newsletters distributed through professional and community organizations. Individuals who expressed interest received the survey link and completed the questionnaire voluntarily.

The inclusion of both university students and working adults was intentional, aiming to capture variability in self-regulatory tendencies across distinct social environments, such as university settings and workplace contexts, where group norms, hierarchical relationships, and interpersonal expectations may differ. Thus, the present design examined whether associations between SOA dimensions and psychological adjustment are observable across heterogeneous adult subgroups rather than within a single occupational or educational group. Because participants were recruited through university courses and personal or social networking channels, the study employed an opportunity/convenience sampling approach. Accordingly, the present participants should be considered an analysis group rather than a representative sample of the Japanese population. The primary aim of this study was to test theoretically grounded associations between culturally socialized self-construal dimensions and psychological adjustment, thereby contributing to theory-driven understanding of mental health processes in contemporary Japanese contexts, rather than to estimate population-level parameters.

At the beginning of the survey, participants viewed an online information sheet explaining the study’s purpose, procedures, voluntary participation, confidentiality protections, and the right to withdraw at any time without penalty. Informed consent was obtained electronically by having respondents check a consent box. The survey was administered via Google Forms, and all items were required to ensure complete responses. Participants completed the questionnaire on either a computer or smartphone, typically within 10–15 min.

A total of 564 individuals accessed the survey and agreed to participate. After excluding cases with missing data or inconsistent response patterns, 422 participants were retained for analysis (effective response rate = 74.82%). All participants were adults residing in Japan. The final sample comprised 315 university students (100 men, 207 women, 8 non-binary/genderqueer) and 107 working adults (62 men, 45 women). Among university students, the mean age was *M* = 19.72 (*SD* = 3.38), with gender-specific means of *M* = 20.40 (*SD* = 5.62) for men, *M* = 19.38 (*SD* = 1.27) for women, and *M* = 19.88 (*SD* = 2.70) for non-binary/genderqueer participants. Among working adults, the mean age was *M* = 46.53 (*SD* = 12.67), with subgroup means of *M* = 46.24 (*SD* = 12.20) for men and *M* = 46.93 (*SD* = 13.42) for women. Across the full sample, the overall mean age was *M* = 26.52 (*SD* = 13.63).

These descriptive statistics are reported solely to characterize the composition of the analysis group and should not be interpreted as estimates of population parameters. Recruitment targets for specific subgroup proportions (e.g., university students vs. working adults) were not pre-specified; accordingly, the final sample composition reflects differential response rates across multiple recruitment channels rather than intentional quota sampling.

The questionnaire additionally included items assessing basic demographic information—gender, age, nationality, occupation, and overseas residence experience—along with the psychological measures described in the following sections.

However, because recruitment relied primarily on university courses and social networking channels, the sample may involve self-selection bias and limited generalizability. Furthermore, the online administration via Google Forms prevented control over the response environment, which may have introduced additional variability in how participants engaged with the survey.

### 2.2. Ethical Considerations

This study was conducted in accordance with the ethical principles of the Declaration of Helsinki and was approved by the Ethics Committee of the Graduate School of Sociology, Toyo University (approval code: P240003). All participants were informed about the purpose of the study, the voluntary nature of participation, their right to withdraw at any time, and the procedures for data handling and anonymization. Informed consent was obtained electronically before the questionnaire began. All data were stored in password-protected files on secure university servers, and no personally identifiable information was collected. The data were used solely for academic research purposes.

### 2.3. Measures

#### 2.3.1. Self-Construal (SOA)

Cultural self-construal was measured using the Japanese version of the Subjective–Objective–Autonomous (SOA) Self-Construal Scale, originally developed by Han, Kim, and Inumiya [20] and validated for Japanese samples by Inumiya et al. [22]. The scale consists of 18 items, forming three subscales: Subjective Self (SS), Objective Self (OS), and Autonomous Self (AS).

All items were rated on a 7-point Likert scale (1 = “does not apply at all,” 7 = “applies very much”). Scores for each subscale were obtained by summing the corresponding items; higher scores indicate a stronger endorsement of each self-construal dimension.

#### 2.3.2. Rosenberg Self-Esteem Scale (SES)

Global self-esteem was assessed using two items from the Japanese version of the Rosenberg Self-Esteem Scale (SES), originally developed by Rosenberg [43] and translated/validated for Japanese samples by Mimura and Griffiths [44].

A shortened form was adopted to reduce respondent burden, as short-form and single-item SES measures have shown strong convergence with the full scale in prior validation studies [45,46].

Participants responded on a 6-point Likert scale (1 = “strongly disagree,” 6 = “strongly agree”). Scores were summed, with higher values reflecting higher global self-esteem.

#### 2.3.3. Subjective Well-Being (SWB)

The Japanese Wave 7 (2022) of the World Values Survey [47] was used to assess subjective well-being via three items assessing life satisfaction, subjective health, and general happiness. Each item was rated on a 6-point scale, and the items were summed to create a composite SWB score; higher scores indicate greater subjective well-being.

#### 2.3.4. General Health Questionnaire-12 (GHQ)

Psychological distress was assessed using the Japanese validated version of the 12-item General Health Questionnaire-12 (GHQ), originally developed by Goldberg and Williams [48] and adapted for Japanese samples by Nakagawa and Daibou [49]. Participants rated each item on a 4-point response scale, indicating the extent to which they had experienced recent symptoms (e.g., concentration problems, depressed mood, sleep disturbance). Following standard GHQ procedures, items were scored using the 0–0–1–1 method, and total scores (0–12) were calculated by summing the recoded responses; higher scores indicate greater psychological distress.

#### 2.3.5. Athens Insomnia Scale (AIS)

Insomnia symptoms were assessed using the Japanese version of the Athens Insomnia Scale (AIS), originally developed by Soldatos et al. [50] and validated for Japanese samples by Okajima et al. [51].

The AIS contains 8 items, each rated on a 4-point scale (0–3), assessing sleep initiation, sleep maintenance, early awakening, overall sleep quality, and daytime functioning. Item scores were summed to yield a total AIS score (0–24), with higher scores indicating more severe insomnia symptoms.

### 2.4. Data Analysis

All analyses were conducted using IBM SPSS Statistics (Version 29.0). Prior to hypothesis testing, internal consistency (Cronbach’s *α*) was calculated for all psychological scales, and descriptive statistics (means and standard deviations) and Pearson’s correlation coefficients were examined for the SOA subscales (SS, OS, AS) and the four psychological adjustment indicators.

To examine the unique predictive effects of cultural self-construal, hierarchical multiple regression analyses were conducted. In each model, age and gender were entered as control variables in Step 1, followed by the three SOA dimensions (SS, OS, AS) in Step 2. Separate regression models were estimated for each dependent variable:

(1) Self-esteem and subjective well-being (positive adjustment indicators);
(2) Psychological distress (GHQ) and insomnia symptoms (AIS) (negative adjustment indicators).

Model fit and effect sizes were evaluated using changes in explained variance (*ΔR*^2^) and standardized regression coefficients (β). Statistical significance was determined using thresholds of *p* < 0.05, *p* < 0.01, and *p* < 0.001. Multicollinearity was checked via variance inflation factors (VIF < 10), and assumptions of normality and homoscedasticity of residuals were examined. Robust standard errors (HC3) were applied when appropriate. For each regression model, *ΔR*^2^, β coefficients, and 95% confidence intervals are reported.

To address the primary aim of this study, all hypothesis testing was conducted using the full sample of Japanese adults, treating the dataset as a single cultural group. Because the student and worker subsamples differed in mean age and social-role contexts, we additionally conducted supplementary subgroup analyses as robustness checks to examine whether the full-sample patterns generalized across these groups. These subgroup analyses were treated as sensitivity checks rather than formal group comparisons, and all main interpretations were based on the full-sample results. Detailed subgroup tables are provided in the Supplementary Materials.

## 3. Results

### 3.1. Descriptive Statistics and Correlations

Internal consistency coefficients for all measures ranged from acceptable to good: Subjective Self (SS: α = 0.67), Objective Self (OS: α = 0.77), and Autonomous Self (AS: α = 0.77); Self-Esteem (SES: α = 0.79); Subjective Well-Being (SWB: α = 0.87); General Health Questionnaire-12 (GHQ: α = 0.87); and Athens Insomnia Scale (AIS: α = 0.80).

Descriptive statistics and bivariate correlations among the study variables are presented in Table 1. SS showed significant positive correlations with SES (*r* = 0.26, *p* < 0.01) and SWB (*r* = 0.18, *p* < 0.05). AS was also positively associated with SES (*r* = 0.16, *p* < 0.01) and SWB (*r* = 0.16, *p* < 0.01). In contrast, OS demonstrated significant negative correlations with SES (*r* = −0.34, *p* < 0.01) and SWB (*r* = −0.17, *p* < 0.01), and significant positive correlations with GHQ (*r* = 0.28, *p* < 0.01) and AIS (*r* = 0.21, *p* < 0.01). AS showed small negative correlations with GHQ (*r* = −0.10, *p* < 0.05) and AIS (*r* = −0.10, *p* < 0.05).

Overall, the SOA self-construal dimensions demonstrated distinct correlational patterns with psychological adjustment indicators, with SS and AS showing positive associations and SWB and OS showing risk-related associations with GHQ and AIS.

### 3.2. Full-Sample Hierarchical Regression Results

To examine the predictive effects of cultural self-construal on psychological adjustment outcomes, a series of hierarchical multiple regression analyses were conducted. Age and sex were entered in Step 1 as control variables, followed by the three SOA dimensions (SS, OS, AS) in Step 2.

#### 3.2.1. Positive Adjustment Indicators

As shown in Table 2, age showed a significant positive effect on self-esteem in Step 1 (β = 0.13, *p* = 0.007). When the SOA dimensions were entered in Step 2, the model showed a significant increase in explained variance (*ΔR*^2^ = 0.15, *p* < 0.001). SS positively predicted SES (β = 0.17, *p* < 0.001). AS showed a significant positive effect (β = 0.12, *p* = 0.010). OS negatively predicted SES (β = −0.26, *p* < 0.001).

As shown in Table 3, adding the SOA dimensions in Step 2 significantly improved model fit for SWB (*ΔR*^2^ = 0.07, *p* < 0.001). SS positively predicted SWB (β = 0.10, *p* = 0.047), AS showed a significant positive association (β = 0.12, *p* = 0.011). and OS emerged as a significant negative predictor (*β* = −0.16, *p* = 0.003). No multicollinearity issues were detected (all VIF values < 2.0).

#### 3.2.2. Negative Adjustment Indicators

As shown in Table 4, age had a significant negative effect on GHQ in Step 1 (β = −0.22, *p* < 0.001). When the SOA dimensions were entered in Step 2, the model showed a significant increase in explained variance (*ΔR*^2^ = 0.06, *p* < 0.001). OS positively predicted GHQ (β = 0.22, *p* < 0.001), AS showed a significant negative effect (β = −0.09, *p* = 0.046), and SS was not significantly associated with GHQ. As shown in Table 5, age showed a significant negative effect on insomnia symptoms in Step 1 (β = −0.10, *p* = 0.043). When the SOA dimensions were entered in Step 2, the model showed a significant increase in explained variance (*ΔR*^2^ = 0.04, *p* < 0.001). OS positively predicted insomnia symptoms (β = 0.19, *p* = 0.001), whereas SS and AS were not significant predictors.

#### 3.2.3. Summary of Core Findings

Across all four hierarchical models, SS and AS consistently emerged as positive predictors of positive psychological adjustment (self-esteem, subjective well-being), whereas OS reliably predicted poorer outcomes on these indicators. For the negative indicators, OS showed consistent positive associations with psychological distress and insomnia symptoms, whereas AS showed weak negative associations and SS did not exhibit significant effects. Overall, the SOA self-construal dimensions demonstrated stable and replicable predictive patterns across psychological outcomes in the full-sample analyses.

### 3.3. Supplementary Subgroup Analyses (Students and Non-Students)

Supplementary subgroup analyses examined whether the full-sample patterns generalized across students and workers. In both groups, SS and AS showed positive associations with SES and SWB, whereas OS demonstrated negative or risk-related associations. The magnitude of OS effects tended to be stronger among students, while AS played a relatively more central role among workers. Notably, the direction of associations remained consistent across both subgroups, indicating that the overall pattern observed in the full sample was stable across role and age contexts. Detailed subgroup regression tables are provided in the Supplementary Materials (Tables S1–S5). These results support the robustness of the full-sample findings.

## 4. Discussion

The primary aim of this study was to clarify how the three dimensions of the SOA model (SS, OS, AS) function psychologically among Japanese adults as a single cultural group. Supplementary subgroup analyses for students and workers were conducted solely to confirm the robustness of the full-sample findings and were not intended as formal group comparisons.

Although the present study employs psychological constructs and self-report measures, its implications are not limited to individual-level processes. Cultural self-construals such as SS, OS, and AS are socially learned and reinforced through education, workplace norms, and everyday social interaction. Therefore, the observed associations with distress and sleep problems have direct relevance for population-level mental health prevention and public health policy.

Consistent with H1, SS and AS were positively associated with self-esteem and subjective well-being. Supporting H2, OS showed negative associations with both positive indicators. Finally, in line with H3, OS was positively associated with psychological distress and insomnia symptoms, whereas AS showed a negative association with psychological distress and a marginal negative association with insomnia symptoms.

Taken together, these findings indicate that SS and AS function as internally oriented psychological resources that promote adaptive well-being, whereas OS consistently operated as a culturally reinforced yet psychologically costly dimension linked to poorer mental health outcomes. These patterns suggest that the SOA model provides a strong framework for understanding within-culture variations in psychological adjustment in contemporary Japan.

Before interpreting the theoretical implications of these findings, it is important to clarify the scope and validity of the conclusions drawn from the present study. Although the participant group was obtained through an opportunity/convenience sampling approach and is not representative of the Japanese adult population, the primary purpose of this research was not to estimate population-level parameters but to examine theoretically grounded associations between culturally socialized self-construal dimensions and multiple indicators of psychological adjustment. From this theory-driven perspective, limited population representativeness does not undermine the analytic validity of the observed relationships. The inclusion of heterogeneous adult subgroups—university students and working adults with diverse ages and social roles—introduced substantial variability in key psychological constructs, which can enhance the robustness of correlational analyses by allowing theoretically meaningful associations to emerge across different social contexts. Moreover, the consistency of the present findings with prior SOA-based research conducted in East Asian contexts further supports the interpretability and credibility of the observed patterns. In addition, the use of well-validated measurement instruments and multivariate analytical approaches to simultaneously examine both positive and negative indicators of psychological adjustment provides confidence that the reported associations reflect substantive psychological processes rather than artifacts of sampling or measurement. Accordingly, while caution is warranted in generalizing the results to the broader Japanese population, the present findings offer a theoretically meaningful and empirically grounded contribution that can inform future research on cultural self-construal and sustainable mental health.

### 4.1. Positive Indicators: SS/AS and Well-Being

Consistent with H1, both SS and AS were positively associated with self-esteem and subjective well-being. As Cross et al. [32] argue, agency-based forms of self-construal enhance well-being even in collectivistic contexts because relying on internal standards fosters a sense of self-efficacy and emotional stability. The present findings align with this view: individuals high in SS and AS—who anchor self-evaluations in personal goals, values, and internally generated intentions—appear better able to maintain positive self-regard and affective well-being [32,35,36].

This pattern also resonates with broader cultural contrasts. In Western settings, moderate self-enhancement and positive illusions can buffer against negative feedback [33], whereas norms of modesty and emotional restraint in East Asia tend to suppress explicit self-positivity and self-esteem [28,42]. Despite such cultural constraints, SS and AS functioned as adaptive psychological resources in the Japanese context. SS, which involves active self-expression and interpersonal initiative, may strengthen self-efficacy and contribute to more positive self-evaluations [38]. AS, characterized by value-based decision-making and reduced reliance on external approval, appears to promote internal consistency and more stable well-being [38,40].

Furthermore, the present findings support prior work indicating that associations between self-construal and well-being in East Asia vary across cultural contexts and measurement approaches, rather than conforming to a uniform “collectivistic” pattern [4,41]. In this regard, the effects of SS and AS underscore the importance of internal standards and flexible self-regulation. Such psychological flexibility—rather than rigid self-consistency—may promote greater emotional stability in shifting cultural environments [32]. This interpretation also aligns with eco-cultural perspectives, which propose that adaptive self-ways must navigate evolving cultural values and social expectations [2,28].

Overall, these results suggest that SS and AS represent internally oriented and flexible forms of self-regulation that support positive psychological functioning in contemporary Japan.

### 4.2. Negative Indicators: OS, Distress, and Sleep Problems

The finding that OS was positively associated with psychological distress and insomnia symptoms suggests that heightened sensitivity to others’ evaluations and social comparison is linked to greater psychological strain and poorer sleep quality. Individuals high in OS tend to base self-evaluations on external standards and regulate their behavior in accordance with perceived expectations and obligations [20,22,23]. Such other-focused self-awareness can amplify self-critical thinking and ruminative tendencies, thereby increasing vulnerability to depression, anxiety, and sleep problems [12,35,36]. This pattern is consistent with Kafetsios [52], who demonstrated that interdependent forms of self-construal may enhance relationship satisfaction in positive emotional contexts but heighten stress responses when negative emotions arise. These findings suggest that individuals high in OS may strive to maintain social harmony while having limited tolerance for negative affect, leading to chronic emotional fatigue and diminished sleep quality.

Evidence from Japan further supports this mechanism. Prior work has shown that higher levels of OS are associated with increased sensitivity to evaluation by others and stronger tendencies toward self-restraint [26,27]. In cultural contexts where “wa” (social harmony), modesty, and emotional restraint are strongly valued, OS-related tendencies—such as other-orientation and suppression of personal desires—may be socially reinforced yet psychologically costly. Uchida and Kitayama [28] noted that East Asian cultural conceptions of happiness emphasize relational harmony over self-realization. The prominence of OS in Japan may reflect this relational orientation, yet the strong normative pressure to maintain harmony may also motivate individuals high in OS to suppress emotional expression and regulate their internal desires to meet others’ expectations. Over time, such suppressive regulation can undermine both mental and physical health.

Japanese display rules that emphasize emotional restraint—particularly in public or interpersonal settings [42]—further intensify these tendencies. Although suppression may yield short-term interpersonal benefits, it can incur long-term physiological costs. This pattern aligns with findings that Japanese individuals tend to exhibit lower nonverbal expressiveness and stronger suppression tendencies than South Koreans [26,27]. Individuals high in OS may therefore prioritize harmony to the extent that they internalize negative emotions rather than expressing them, contributing to psychological fatigue and poorer sleep. In cultures characterized by strong suppression norms, such as Japan, the “short-term benefits” of suppression can readily transform into “long-term costs,” helping explain why OS is consistently associated with heightened psychological vulnerability [8,10,11].

Finally, the present study found that age was positively associated with self-esteem and negatively associated with psychological distress and insomnia symptoms, suggesting that psychological health tended to stabilize with increasing age, although its association with subjective well-being was not significant. These patterns are consistent with socioemotional selectivity theory, which proposes that older adults exhibit more effective emotion regulation and reduced reactivity to negative stimuli [53], as well as with developmental research indicating that self-esteem increases from adolescence into mid-adulthood [31]. Taken together, these findings suggest that the effects of cultural self-construal observed in this study reflect stable psychological tendencies that are not reducible to cohort or developmental stage.

## 5. General Discussion

This study examined how the three dimensions of the SOA model—Subjective Self (SS), Objective Self (OS), and Autonomous Self (AS)—jointly predict both positive and negative aspects of psychological adjustment among Japanese adults. Across all indicators, a consistent dual pattern emerged: SS and AS functioned as psychological resources that supported higher self-esteem and subjective well-being, whereas OS was reliably associated with greater psychological distress and insomnia symptoms. These findings indicate that internally oriented selfways facilitate adaptive adjustment even in a culture that traditionally emphasizes modesty and emotional restraint. Conversely, OS—a culturally normative and socially desirable selfway in Japan—nonetheless may operate as a vulnerability pathway linked to poorer mental health. This apparent tension suggests that the psychological implications of OS cannot be fully understood through cultural norms alone and may instead reflect deeper cultural, structural, and regulatory dynamics. The following sections integrate these findings with broader theoretical perspectives to clarify the mechanisms through which cultural self-construal promotes or undermines sustainable mental health in contemporary Japanese society.

### 5.1. Theoretical Implications

The present findings provide several theoretical insights into how cultural self-construal operates within the contemporary Japanese context. First, the results illustrate that the psychological meaning of OS is shaped not only by interpersonal motives but also by broader eco-cultural dynamics. According to Kitayama and Salvador [2], long-standing cultural practices can reduce “meaning flexibility” and lead to norm-based behaviors being rigidly internalized. In Japan—where humility, emotional restraint, and inter-personal sensitivity have been historically reinforced—OS-related tendencies such as vigilance to evaluation and self-suppression may become fixed behavioral meanings rather than contextually adaptive strategies [5,26,27,42].

This interpretation is also consistent with evidence that Japan is a culturally “tight” society characterized by strong normative expectations and low tolerance for deviance, as demonstrated by Gelfand et al. [54]. Under such conditions, OS-like behaviors become obligatory rather than optional, which may intensify their psychological cost. Second, the present findings highlight a growing misalignment between traditional cultural norms and contemporary social structures. Historically, values such as “wa” (harmony), modesty, and self-restraint aligned well with OS and supported relational stability. However, modern Japanese society increasingly emphasizes autonomy, individual achievement, and expressive communication—pressures intensified by contemporary work structures and digital media environments. Prior work supports this emerging value conflict. Uchida and Kitayama [28] argued that relational harmony, while culturally valued, can function as an external pressure that suppresses subjective well-being. Similarly, Park et al. [4] found that although emotional suppression is normatively encouraged, it is paradoxically associated with reduced well-being in Japanese adults. The present pattern—OS predicting greater psychological distress and insomnia symptoms—appears consistent with these broader cultural tensions.

Taken together, these implications suggest that OS may have functioned adaptively under traditional relational ecologies but becomes psychologically costly in a pluralistic society that increasingly demands autonomy and self-direction. In contrast, SS and AS—with their emphasis on internal standards, agency, and value-based self-regulation—appear to function as psychological resources that remain beneficial even within a culture that discourages overt self-assertion. This interpretation aligns with research showing that agency-based self-views promote subjective well-being even within East Asian cultural contexts, as demonstrated by Cross et al. [32] and Ryu, Kim, & Han [38]. These findings reinforce the perspective that cultural self-construal should be understood not as a fixed cultural type but as a dynamic psychological system whose adaptiveness depends on cultural tightness, value congruence, and socio-historical change. Although OS aligns well with culturally prescribed norms in Japan—such as “wa”, modesty, and emotional restraint—the present findings also suggest that OS may conflict with individuals’ internal psychological needs for autonomy and authentic self-expression. In this sense, OS may represent a culturally adaptive yet psychologically costly selfway, pointing to the possibility of a latent misalignment between cultural expectations and personal regulatory needs. However, this interpretation remains provisional, as the present study did not directly measure cultural fit; future research is needed to empirically test when and for whom this misalignment emerges.

### 5.2. Practical Implications

The present findings also carry practical implications for supporting sustainable mental health in contemporary Japanese society. Although OS is culturally normative and socially rewarded—particularly in contexts that emphasize harmony and emotional restraint—its psychological costs suggest the need for interventions that supplement, rather than replace, OS-oriented tendencies. Programs that strengthen internally guided psychological resources, such as agency (SS) and autonomy (AS), may help buffer the emotional vulnerabilities associated with heightened evaluative vigilance. For example, psychoeducational interventions can encourage individuals to identify personal values, practice self-initiated decision-making, and develop problem-focused coping strategies that rely less on external approval.

In cultures where emotional suppression is socially expected, direct promotion of emotional expression may be culturally incongruent. Instead, culturally compatible strategies—such as low-intensity or indirect emotional disclosure (“soft expression”)—may provide a feasible means of reducing the physiological burden of chronic suppression without threatening relational harmony [3,30]. Mindfulness-based practices and relaxation-oriented routines may further mitigate the stress–arousal cycle associated with OS by supporting parasympathetic activation, improving emotion regulation, and enhancing sleep quality [18].

At the organizational and educational levels, environments that value both relational sensitivity and internal self-guidance may help reduce the tension experienced by high-OS individuals. Workplaces and schools that adopt flexible communication norms, encourage reflective dialogue, and soften rigid expectations for emotional restraint may be particularly effective. Because OS is reinforced through daily socialization processes, preventive approaches introduced early—such as value clarification, social–emotional learning, and adaptive coping training—may play a vital role. More broadly, the findings suggest that sustainable mental health in Japan may depend on recognizing the dual nature of OS: socially adaptive yet psychologically costly. Public mental health strategies that acknowledge individual differences in self-construal and reduce excessive normative pressures to maintain harmony may contribute to more balanced forms of self-regulation. Supporting multiple culturally viable pathways to well-being—rather than enforcing a single relational ideal—may allow individuals to navigate the competing demands of modern Japanese society with greater resilience. At the policy level, these findings support public mental health strategies that reduce excessive social-evaluative pressure and expand culturally acceptable coping resources through population-wide education and organizational practices.

### 5.3. Limitations and Future Directions

Several limitations of the present study should be acknowledged. First, the cross-sectional design prevents strong causal inferences regarding the directionality of the relationships between SOA dimensions and psychological adjustment. Longitudinal and experimental studies are needed to clarify how SS, OS, and AS prospectively shape trajectories of well-being, psychological distress, and insomnia symptoms over time. Second, all variables were assessed via self-report, raising the possibility of shared method variance and response biases. Future research would benefit from incorporating multi-informant reports, behavioral indicators, and physiological or sleep-based measures to more robustly capture psychological and health outcomes. Third, the sample was not nationally representative and consisted of Japanese adults from specific demographic backgrounds, which may limit the generalizability of the findings. Replication with more diverse samples—across regions, age groups, and socioeconomic strata—would help determine the extent to which the present patterns hold within the broader Japanese population and across East Asian societies.

In addition, several measurement-related limitations should be noted. The internal consistency of the SS subscale was relatively modest, suggesting that further refinement of the scale or the use of alternative indicators of agency-based self-construal may be warranted. Moreover, self-esteem was assessed using a two-item short form of the Rosenberg Self-Esteem Scale. Although brief SES variants have shown good convergence with the full scale in prior validation work, the limited number of items may have reduced construct coverage and comparability with studies using the full instrument. Finally, the effect sizes observed in this study were modest (*ΔR*^2^ = 0.04–0.15). However, this range is consistent with prior work showing that individual differences in self-construal typically account for small but reliable proportions of variance in psychological functioning [26,38,41]. Thus, while the present findings do not indicate large individual-level effects, they remain theoretically meaningful and are comparable to effect sizes repeatedly reported in the cultural self-construal literature.

A key conceptual limitation is that the present study did not directly assess cultural fit—that is, the degree of congruence or mismatch between individuals’ self-construal and the cultural norms they perceive in their social environment. Although our interpretations drew on cultural-fit frameworks to explain why OS may be socially normative yet psychologically costly in Japan, these explanations remain inferential without direct measurement. Future studies should explicitly operationalize cultural fit using indicators such as values congruence, perceived normative expectations, or emotional fit with culture. Such measures would help clarify the conditions under which OS functions as a cultural resource versus a psychological risk factor. Moreover, examining cultural fit as a mediator or moderator would allow for a more precise understanding of how individual-level self-construal interacts with culturally prescribed regulatory norms to shape mental health outcomes.

Finally, the present analyses were correlational and did not incorporate potential mediating and moderating processes that may account for the links between cultural self-construal and mental health. Future research should investigate mechanisms such as rumination, self-efficacy, coping strategies, and emotion regulation as potential mediators, and consider moderators such as age, gender, situational context, or cultural fit.

## 6. Conclusions

The present study applied the SOA model of cultural self-construal to the Japanese context and demonstrated that the three dimensions—SS, OS, and AS—show distinct and theoretically meaningful associations with mental health. SS and AS consistently emerged as psychological resources that supported higher self-esteem and subjective well-being, highlighting the adaptive value of internally guided self-regulation even in a cultural environment that discourages overt self-assertion. In contrast, OS appeared to function as a risk-related dimension, predicting greater psychological distress and insomnia symptoms despite being culturally normative and socially reinforced in Japan. This pattern provides empirical insight into the OS paradox—namely, how a culturally desirable selfway can nevertheless impose psychological costs under conditions of strong normative pressure.

By jointly examining positive and negative indicators, this study demonstrates that cultural self-construal has dual pathways that can either support or undermine sustainable mental health. These findings refine conventional views of collectivistic adjustment by showing that psychological resilience in contemporary Japan is better predicted by internal standards (SS, AS) than by other-focused regulatory tendencies (OS). Taken together, the results underscore the importance of considering both cultural norms and individual psychological needs when evaluating mental health in culturally tight societies such as Japan. Future work integrating cultural fit and dynamic regulatory processes will further clarify how cultural selfways contribute to sustainable well-being across diverse cultural contexts.

## Figures and Tables

**Table 1:** Descriptive Statistics and Correlations Among Study Variables.

	*M*	*SD*	1	2	3	4	5	6	7
1 SS	21.78	5.57	―						
2 OS	27.19	6.23	−0.319 **	―					
3 AS	27.46	5.96	0.206 **	−0.049	―				
4 SES	7.47	1.08	0.262 **	−0.339 **	0.163 **	―			
5 SWB	11.29	2.89	0.184	−0.173 **	0.156 **	0.593 **	―		
6 GHQ	4.41	3.38	−0.096 *	0.279 **	−0.104 *	−0.486 **	−0.566 **	―	
7 AIS	14.53	3.91	−0.049	0.209 **	−0.097 *	−0.342 **	−0.433 **	0.550 **	―

Note. SS = Subjective Self; OS = Objective Self; AS = Autonomous Self; SES = Self-esteem; SWB = Subjective well-being; GHQ = General Health Questionnaire-12; AIS = Athens Insomnia Scale. * *p* < 0.05, ** *p* < 0.01 (two-tailed).

**Table 2:** Hierarchical Regression Analysis Predicting Self-Esteem Scores.

Variables	β	t	*p*-Value	VIF
age	0.11	2.34	0.020 *	1.15
sex	−0.01	0.49	0.624	1.08
SS	0.17	3.47	<0.001 ***	1.22
OS	−0.26	−5.19	<0.001 ***	1.22
AS	0.12	2.58	0.010 **	1.05

*R*^2^ = 0.17; *ΔR*^2^ = 0.15; Note. * *p* < 0.05, ** *p* < 0.01, *** *p* < 0.001.

**Table 3:** Hierarchical Regression Analysis Predicting Subjective Well-Being Scores.

Variables	β	t	*p*-Value	VIF
age	−0.06	−1.16	0.247	1.15
sex	0.06	1.24	0.217	1.08
SS	0.10	1.20	0.047 *	1.22
OS	−0.16	−3.02	0.003 **	1.22
AS	0.12	2.54	0.011 *	1.05

*R*^2^ = 0.07; *ΔR*^2^ = 0.07; Note. * *p* < 0.05, ** *p* < 0.01.

**Table 4:** Hierarchical Regression Analysis Predicting General Health Questionnaire-12 Scores.

Variables	β	t	*p*-Value	VIF
age	−0.18	−3.65	<0.001 ***	1.16
sex	0.03	0.52	0.605	1.08
SS	−0.03	−0.55	0.581	1.22
OS	0.22	4.34	<0.001 ***	1.22
AS	−0.09	−2.00	0.046 *	1.05

*R*^2^ = 0.19; *ΔR*^2^ = 0.06; Note. * *p* < 0.05, *** *p* < 0.001.

**Table 5:** Hierarchical Regression Analysis Predicting Athens Insomnia Scale Scores.

Variables	β	t	*p*-Value	VIF
age	−0.10	−2.03	0.043 *	1.15
sex	−0.02	−0.39	0.697	1.08
SS	0.02	0.30	0.761	1.22
OS	0.19	3.61	0.001 ***	1.22
AS	−0.09	−1.91	0.056	1.05

*R*^2^ = 0.06; *ΔR*^2^ = 0.04; Note. * *p* < 0.05, *** *p* < 0.001.

## Data Availability

The data presented in this study are available on request from the corresponding author due to ethical restrictions and participant confidentiality.

## Supplementary Materials

The following supporting information can be downloaded at https://www.mdpi.com/article/10.3390/ijerph23020197/s1: Table S1a,b: Correlation matrices among main study variables for university students and working adults, respectively. Table S2a,b: Hierarchical regression analyses predicting self-esteem (SES) for university students and working adults. Table S3a,b: Hierarchical regression analyses predicting subjective well-being (SWB) for university students and working adults. Table S4a,b: Hierarchical regression analyses predicting psychological distress (GHQ) for university students and working adults. Table S5a,b: Hierarchical regression analyses predicting insomnia symptoms (AIS) for university students and working adults.

## Abbreviations

SOA	Subjective–Objective–Autonomous Model
SS	Subjective Self
OS	Objective Self
AS	Autonomous Self
SES	Self-Esteem Scale
SWB	Subjective Well-Being
GHQ	General Health Questionnaire
AIS	Athens Insomnia Scale
